# Three-dimensional reconstruction of the suborbicularis oculi fat and the infraorbital soft tissue

**DOI:** 10.1016/j.jpra.2018.01.001

**Published:** 2018-01-31

**Authors:** T. Sandulescu, T. Blaurock-Sandulescu, H. Buechner, E.A. Naumova, W.H. Arnold

**Affiliations:** Department of Biological and Material Sciences in Dentistry, School of Dentistry, Faculty of Health, Witten/Herdecke University, Germany

**Keywords:** Suborbicularis oculi fat, SOOF plastic surgery, Subcutaneous musculoaponeurotic system, SMAS, 3D reconstruction, Infraorbital soft tissue

## Abstract

The aim of this study was to reveal the histomorphological connections among the suborbicularis oculi fat (SOOF), the orbicularis oculi muscle (OOM), the superficial musculoaponeurotic system (SMAS), the infraorbital fat and the skin.

Full graft tissue blocks of the infraorbital region with the skin, SMAS, OOM and SOOF were collected post mortem from one female and two male formalin-fixed body donors. Serial histological sections were made, stained and digitized. Digitalization and three-dimensional (3D) reconstruction of the histological meshwork were performed.

SOOF was revealed as a fibro-adipose tissue underlying the OOM, which was strictly separated from the intraorbital fat pad by the orbital septum. SOOF, OOM and SMAS were connected by fibrous septa derived from the SOOF, traversing the OOM with division into multiple muscular bundles, continuing above the muscular plane by forming the SMAS and ending with skin insertion. In the infraorbital region, two different types of SMAS bordering the infraorbital fold have been recognized. Muscle cells have been demonstrated in the SMAS fibrous septa of both SMAS types.

Together with the OOM, the SMAS and the skin, SOOF forms an anatomical functional unit. Muscular contraction of the OOM could be transferred by the SMAS to the skin level, producing periorbital mimic expression. The 3D reconstruction facilitates the comprehension of the morphological structure, its connections and space correlations in the infraorbital area. The morphological and topographical peculiarities of the infraorbital structures make it possible to conclude that surgical interventions in this area need to be elaborated and individualized.

## Introduction

The suborbicularis oculi fat (SOOF) was defined as a fat pad lying supraperiosteally beneath the orbicularis oculi muscle (OOM) and below the lateral half of the infraorbital rim above the zygoma.^1^ The hockey stick-shaped head of SOOF is located at the inferolateral side of the orbit within a range of +15 degrees medial and −87 degrees lateral to a caudal vertical mid-pupillary line.^2^ It has an average horizontal length of 48 mm and an average vertical height of 27 mm.^2^

Previous studies described that the midfacial fat compartments (malar and SOOF) are connected to the orbicularis oculi muscle and the facial bone by the superficial musculoaponeurotic system (SMAS).^3^ The lack of a clear definition of the SMAS structure allowed the authors to conclude that SMAS inserts in the orbicularis oculi muscle (OOM) connection to the facial bone.^3^, ^4^ Recent investigations described the SMAS as a three-dimensional meshwork connecting the mimic musculature to the skin with communicating fibro-muscular compartments enveloping fat pads.^5^

The anatomy of the periorbital fat pads, especially the SOOF, is well described, and the connection of these structures to the periorbital and cheek SMAS raised interest among oculoplastic reconstructive and rejuvenating surgeons.^1^, ^6^, ^7^ The term SOOF was coined in clinical practice related to the lower eyelid blepharoplasty, where it was resected to avoid contour defects.^1^ However, the SOOF terminology has not yet been implemented in Nomina Anatomica.^8^

The surgical management of chronic facial palsy includes, among other problems, the reposition of the SOOF, which helps elevate the overlying and the lower eyelid tissues because they are connected to each other.^9^ SOOF lifting procedures are thus used as additional surgical maneuver in the correction of the cicatricial lower eyelid ectropion, aging mid-face ptosis and chronic facial palsy to achieve adequate corneal protection and improve the aesthetic facial affect.^6^, ^9^ Furthermore, SOOF plication and suspension are well-described surgical techniques for midfacial rejuvenation.^1^, ^10^ However, these procedures have shown a better long-term effect in congenital cases than in facial palsy, where the subsequent drop is greater due to the heavy tissue.^6^

Nevertheless, the lack of a clear definition of SOOF has allowed controversial opinions and interpretation concerning the anatomical location and morphology of this structure.^2^, ^11^

The aim of this study was to perform histomorphological analysis and three-dimensional (3D) reconstruction of the meshwork of the SOOF structure and its connection to the SMAS, the OOM, the intraorbital fat and the skin. The hypothesis of this investigation was that SOOF is a fat pad with morphological architecture that is similar to that of the SMAS underlying the OOM.

## Methods

Full graft tissue blocks of the skin, SMAS, OOM and SOOF of the lateral infraorbital region were collected post mortem from two male (61 and 80 years old) and one female (80 years old) body donors and fixed in 4.5% formaldehyde. The cadavers were provided by the Department of Anatomy II, Friedrich-Alexander-Universität Erlangen-Nürnberg and were official testamentary donations of volunteers to the Department for the anatomical student course for medical and dental students and for medical research purposes. The study was carried out according to the regulations of the WMA Declaration of Helsinki in its present form from 2013. The donor sites showed no visible scars or tissue damage, and the medical history revealed no surgical intervention or radiation of the head and neck area.

**Figure 1:**
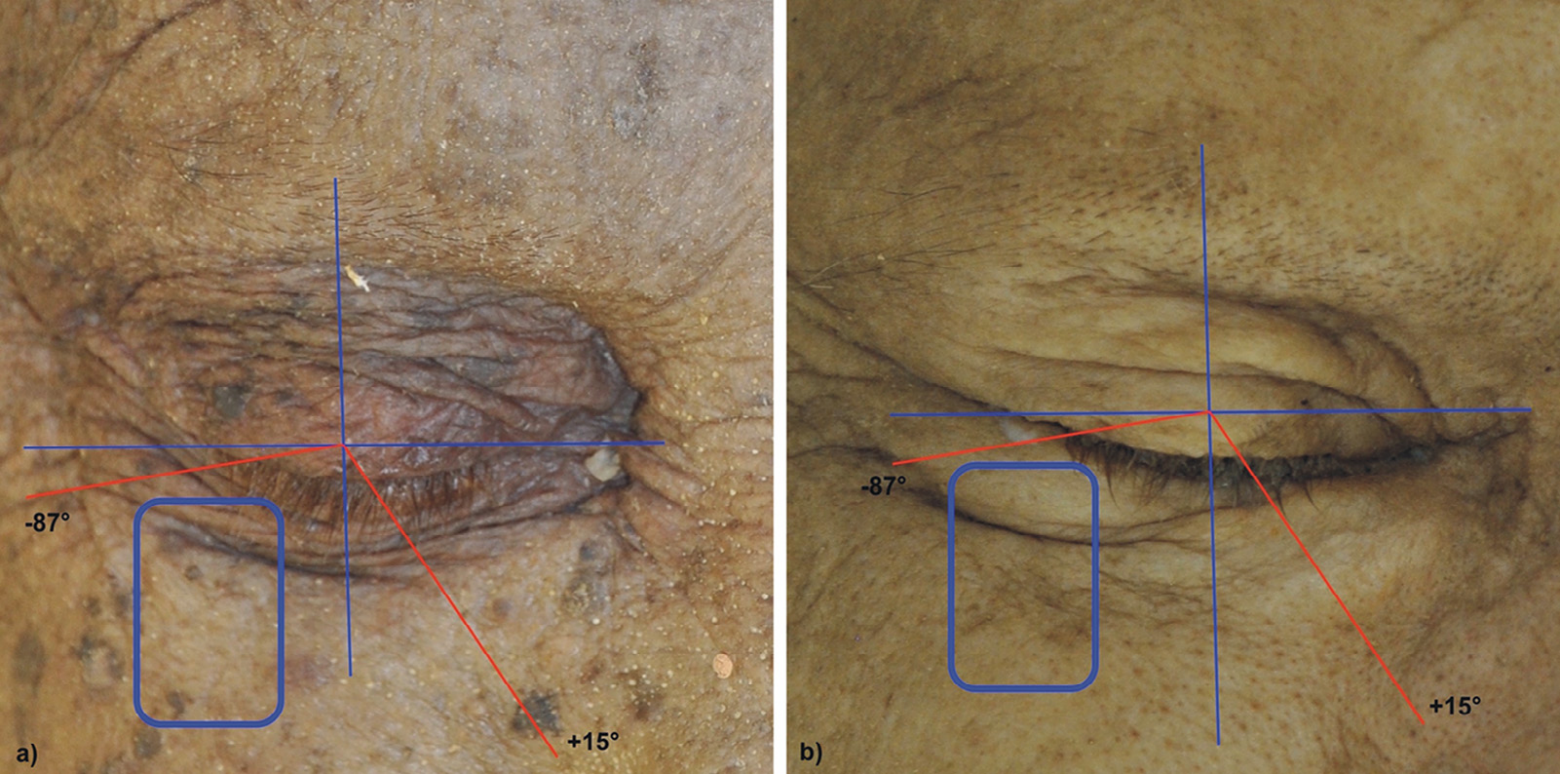
Body donors' sides: a) female and b) male.

SOOF was localized macroscopically by the method described by Hwang et al. (2007), where the tissue blocks were removed (Figure 1).^2^

### Histological analysis

After fixation in 4.5% formaldehyde, the 1×2×1 cm^3^ tissue blocks were embedded in paraffin, and serial histological sections in the vertical plane were cut with a thickness of 5 µm. Every section was collected, and every 10th section was stained with Azan. Photomicrographs of the section were taken using a Nikon D7000 camera (Nikon, Tokyo, Japan) with a resolution of 12 megapixels. In addition, the sections were studied with a Leitz DMRB microscope (Leica, Wetzlar, Germany), and additional micrographs were taken.

### 3D reconstruction

The 3D reconstruction and rendering were performed using AutoCAD 2013 (Autodesk, Munich, Germany). The photographs of the male and female sections were consecutively imported into AutoCAD 2013 and superimposed according to the best fit method. The outlines of the relevant structures were then digitized, each in separate layers. A total of 123 sections for the female and 106 for both male body donors were digitized. Digitizing a single section required between 15 and 25 minutes, depending on the complexity of the traced structures. A 3D meshwork wire frame image was created from each structure. By freezing or thawing single structures (electronic dissection),^12^ the 3D architecture of SOOF structures and its relation to the OOM, the SMAS, the intraorbital fat and the skin were demonstrated. The 3D wire-frame mesh was imported into the 3D Studio (Autodesk, Munich, Germany) computer program, rendered into models and visualized from different angles. Additionally animated videos were rendered with 3D Studio.

## Results

### Histological analysis

**Figure 2:**
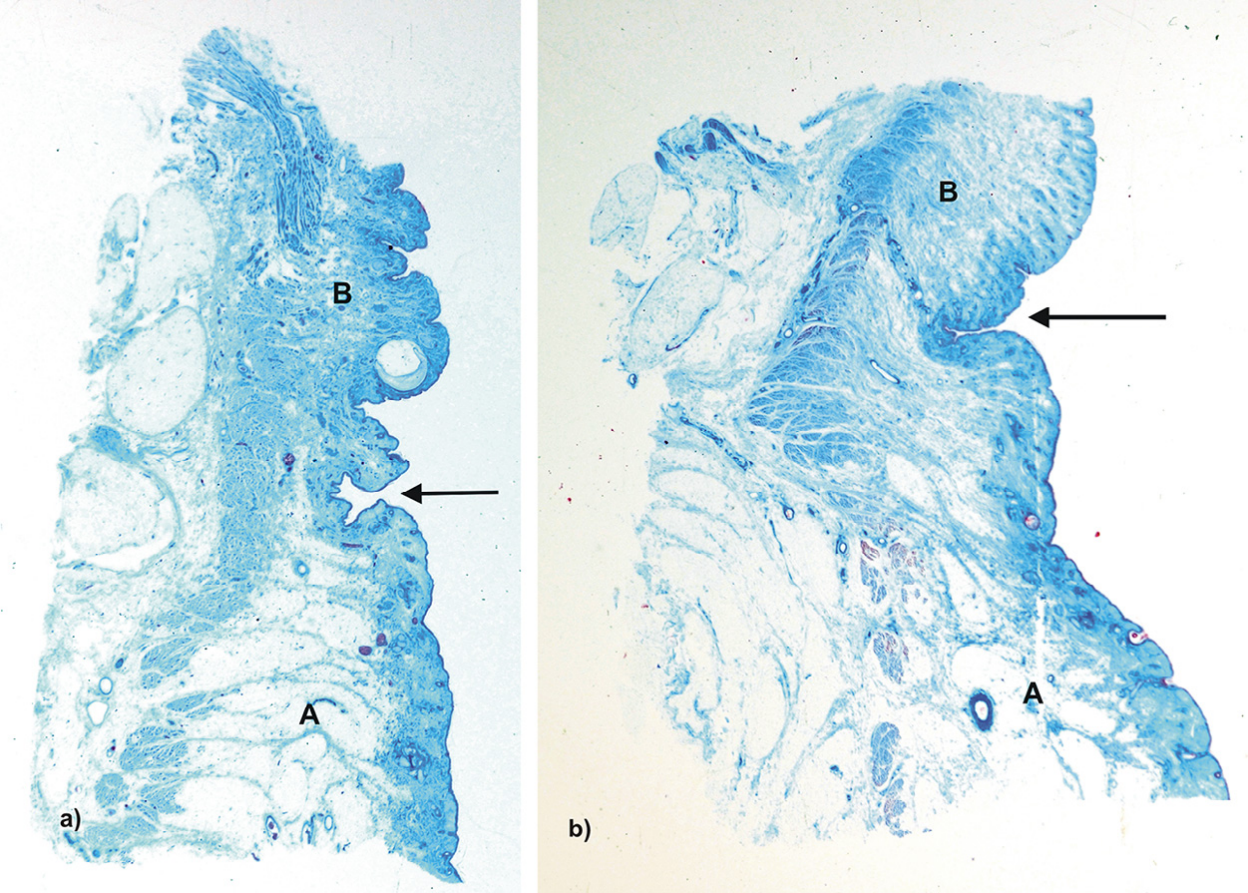
Microphotographic overview of the histological section of the a) specimen one and b) specimen two. A = infraorbital area; B = lower eyelid area. The infraorbital fold is marked with an arrow. A change of the SMAS morphology cranial and caudal to the infraorbital fold is visible.

Macroscopically all three specimens (female: specimen one; male: specimen two and three) presented a prominent infraorbital fold (Figure 1). The histological analysis of the tissue blocks removed from the tissue located at the inferolateral side of the orbit demonstrated the existence of SMAS and SOOF tissue with different morphologies between the specimens (Figure 2).

#### SOOF

**Figure 3:**
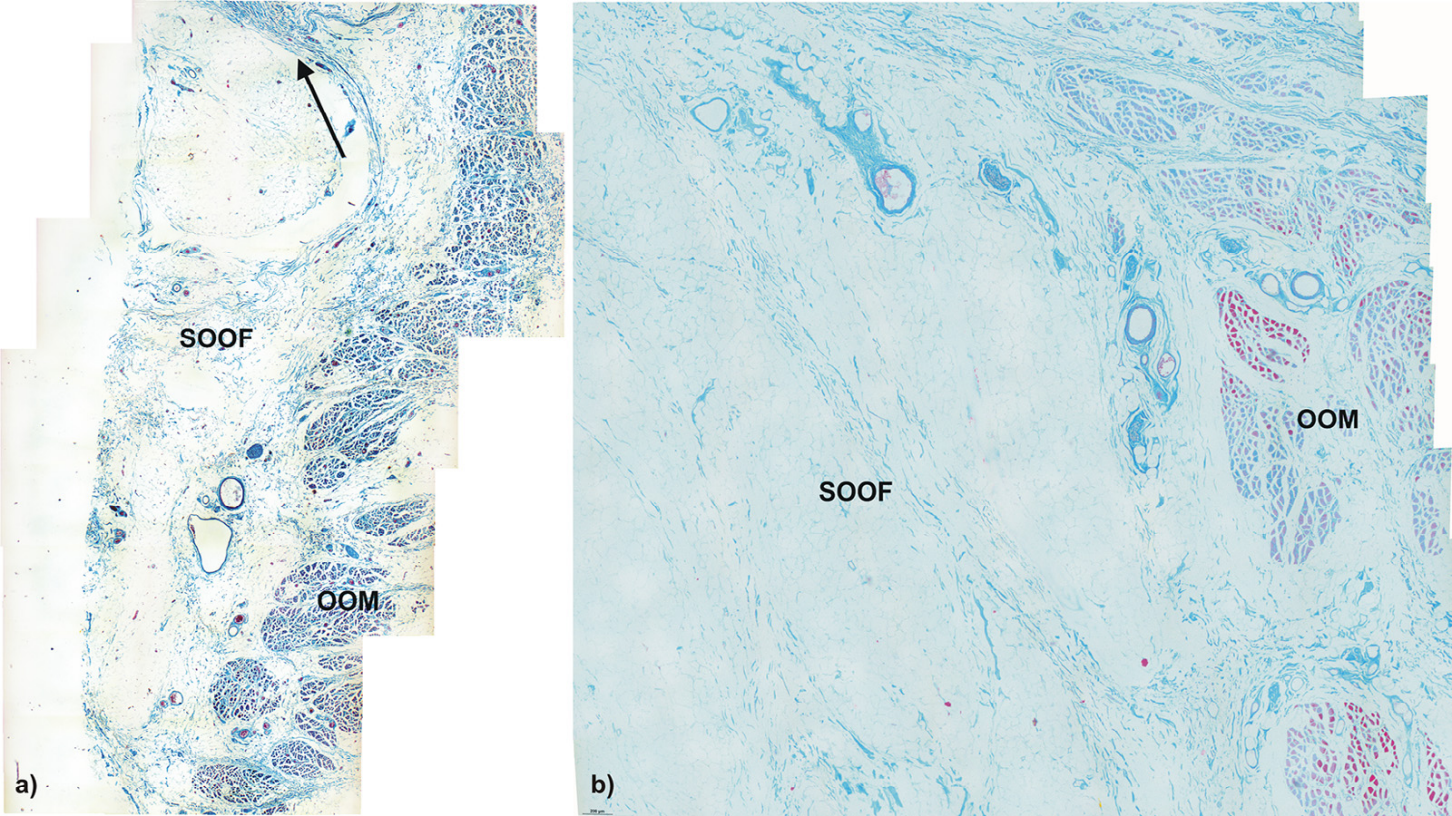
Microphotographic overview of the histological SOOF section lying deep to the OOM from the a) specimen one and b) specimen two. The arrow indicates the septum orbitale.

**Figure 4:**
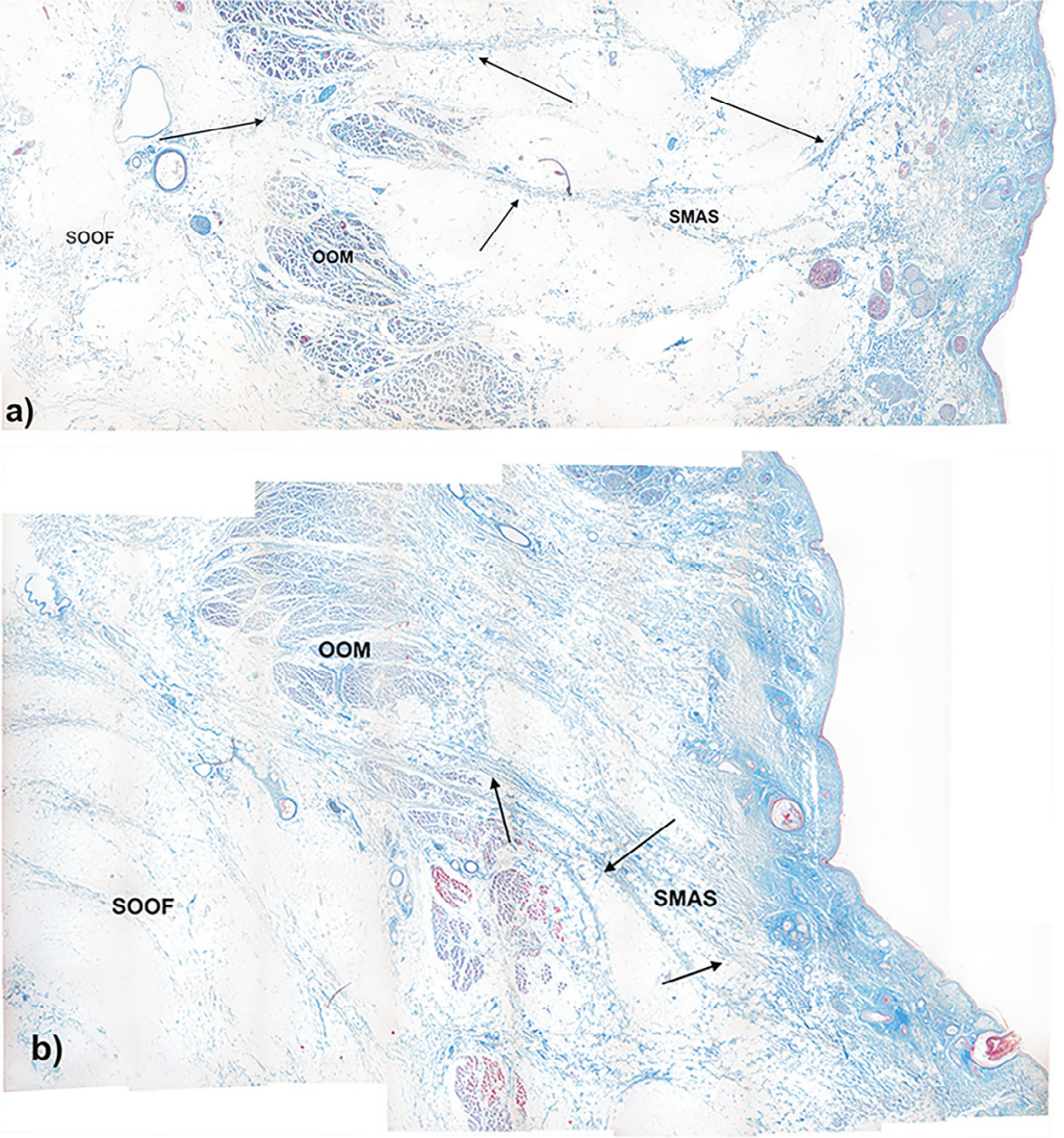
Microphotographic overview of the connection between SOOF, the OOM and SMAS. The fibrous septa (arrows) divide the OOM belly into several muscular tangles: a) specimen one; b) specimen two.

SOOF was identified as a fat pad underlying the OOM. All three specimens had similar morphological SOOF architecture consisting of fibrous septa parallel aligned to the OOM fibers enveloping fat pads (Figure 3). Microscopically, the cranial border of the SOOF did not cross the infraorbital fold level. The bounding fibrous septa spread to the superficial lying OOM, dividing its belly into several muscular tangles. The fibrous septa continued superficial to the OOM, forming the infraorbital SMAS fibro-muscular septa (Figure 4). There was a direct connection between the SOOF and the SMAS. Compared to the SMAS fibers, the SOOF septa were smoother and aligned parallel to the OOM fibers. The microscopic analysis of the SOOF architecture showed specimen-specific differences concerning its volume. The SOOF fibers in all specimens showed similar microscopic architectures.

#### SMAS

The histological analysis of the tissue blocks removed from the lateral infraorbital region demonstrated the existence of the SMAS tissue between the OOM and the skin along the entire length of the tissue block.

**Figure 5:**
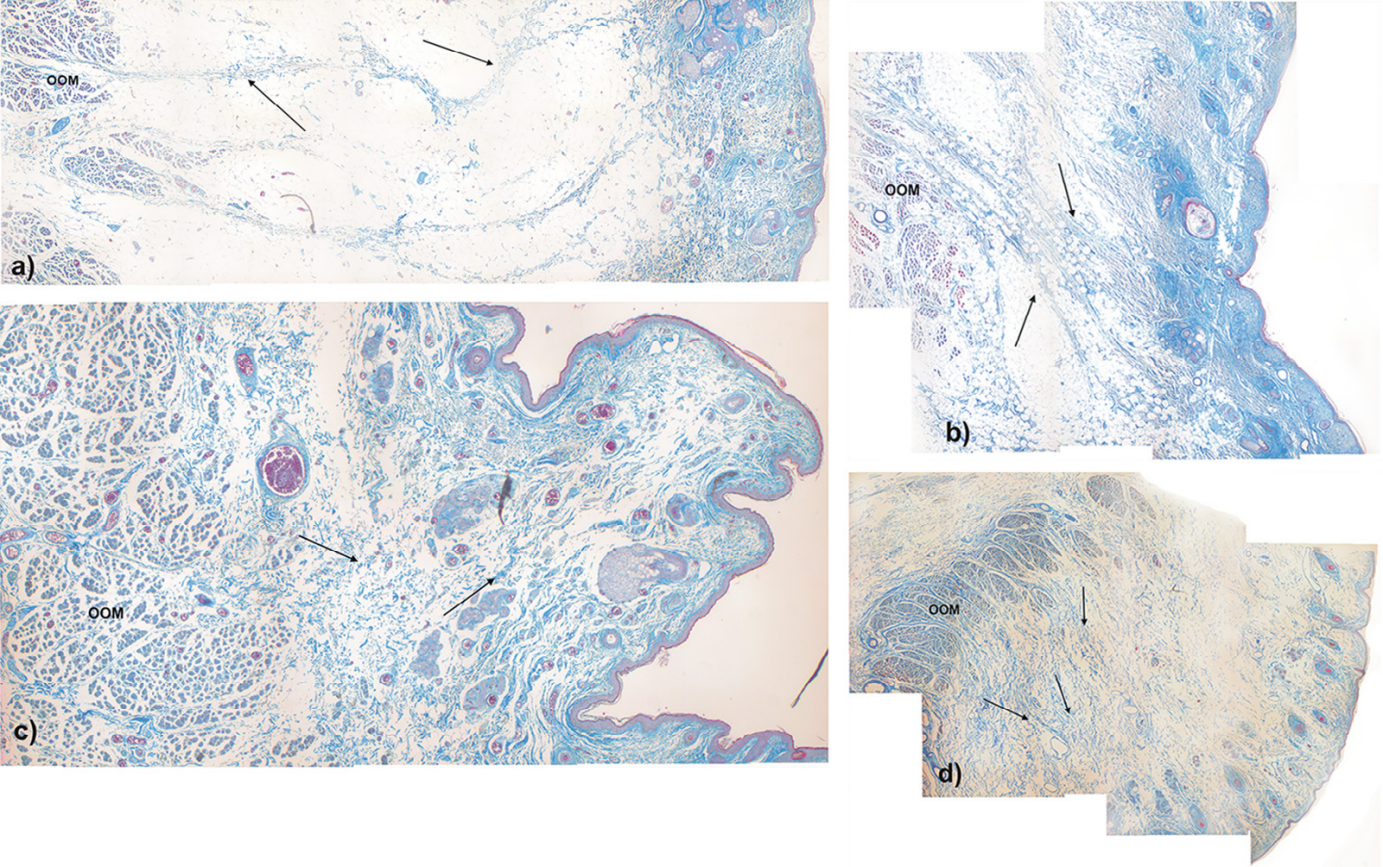
Microphotographic overview of the histological SMAS section of the infraorbital area: a) specimen one, b) specimen two and lower eyelid regions: c) specimen one, d) specimen two showing the fibro-muscular SMAS septa connecting the OOM fiber bundles to the skin.

**Figure 6:**
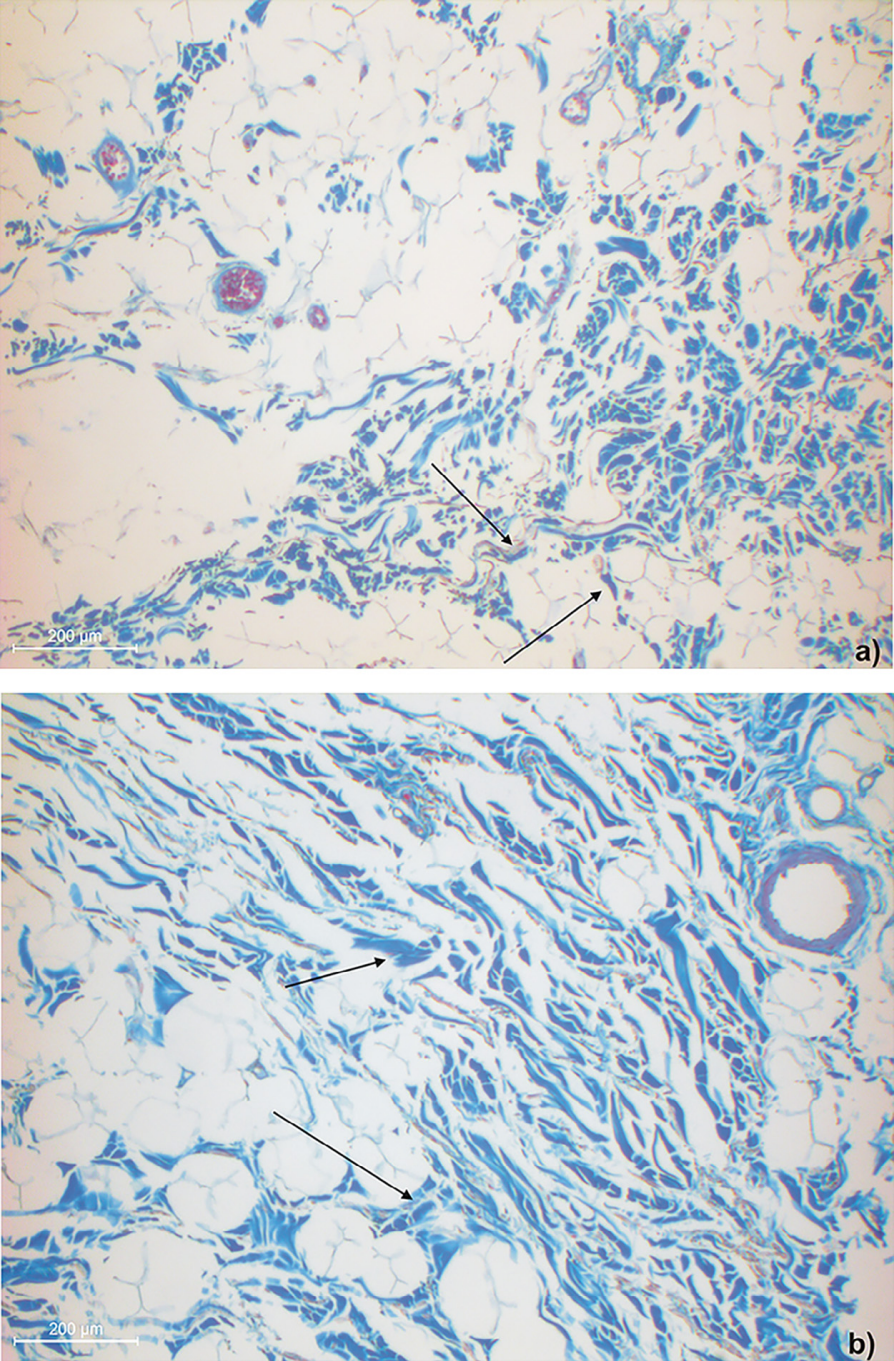
Higher magnification of the fiber insertion inferior to the infraorbital fold into the dermis showing muscle cells in the a) specimen one and b) specimen two.

**Figure 7:**
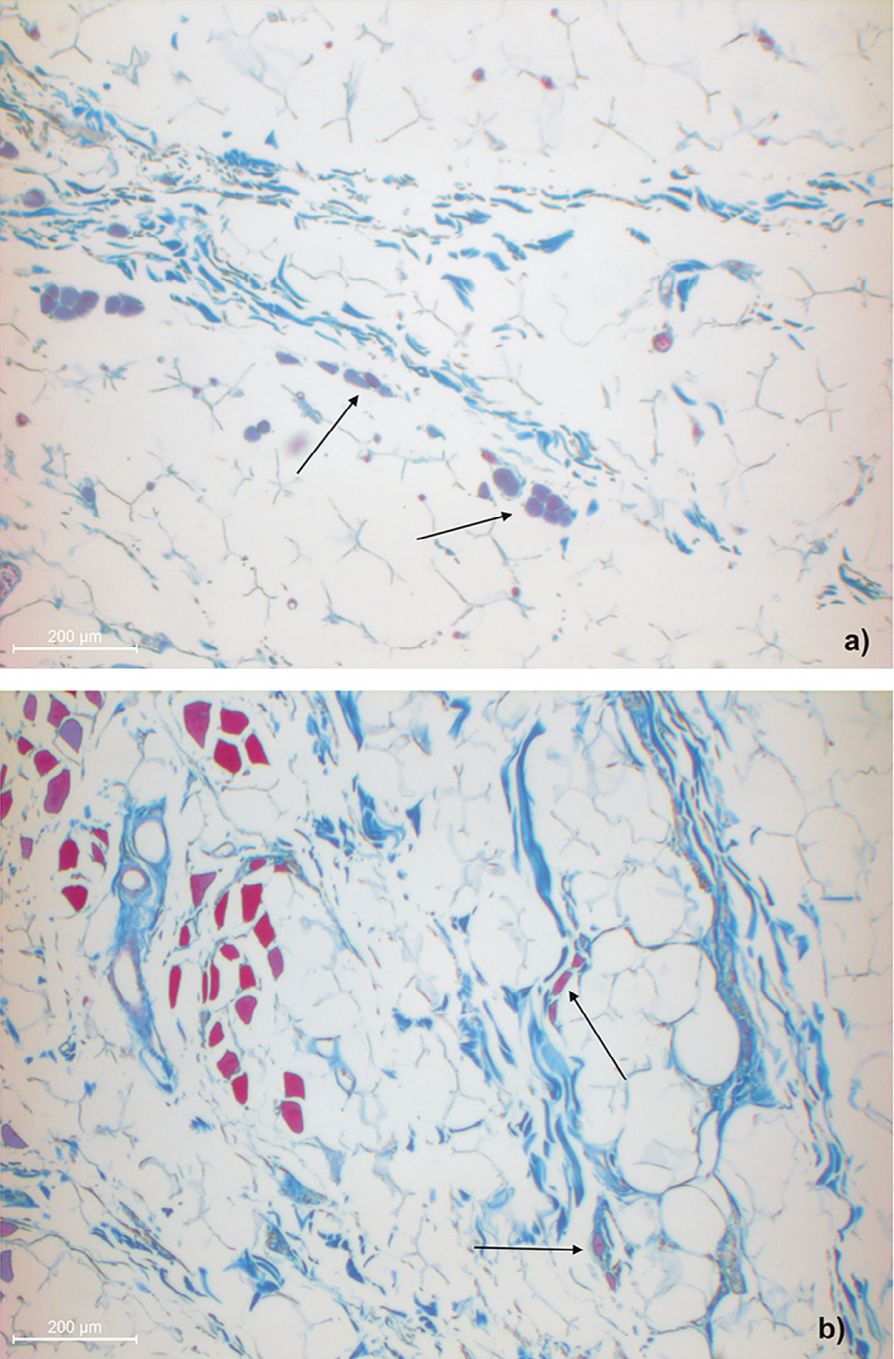
Higher magnification of the fiber insertion inferior to the infraorbital fold into the OOM showing isolated muscle cells in the SMAS septa of the a) specimen one and b) specimen two.

Microscopically, there were two different SMAS architectures cranial and caudal to the infraorbital fold (Figure 5). Caudal to the infraorbital fold, SMAS fibers showed a regular architecture with parallel-running septa connecting the OOM to the skin (Figure 5a and 5b). The fibrous septa of all three specimens contained muscular fibers at the docking areas to the OOM and to the skin (Figure 6, Figure 7).

**Figure 8:**
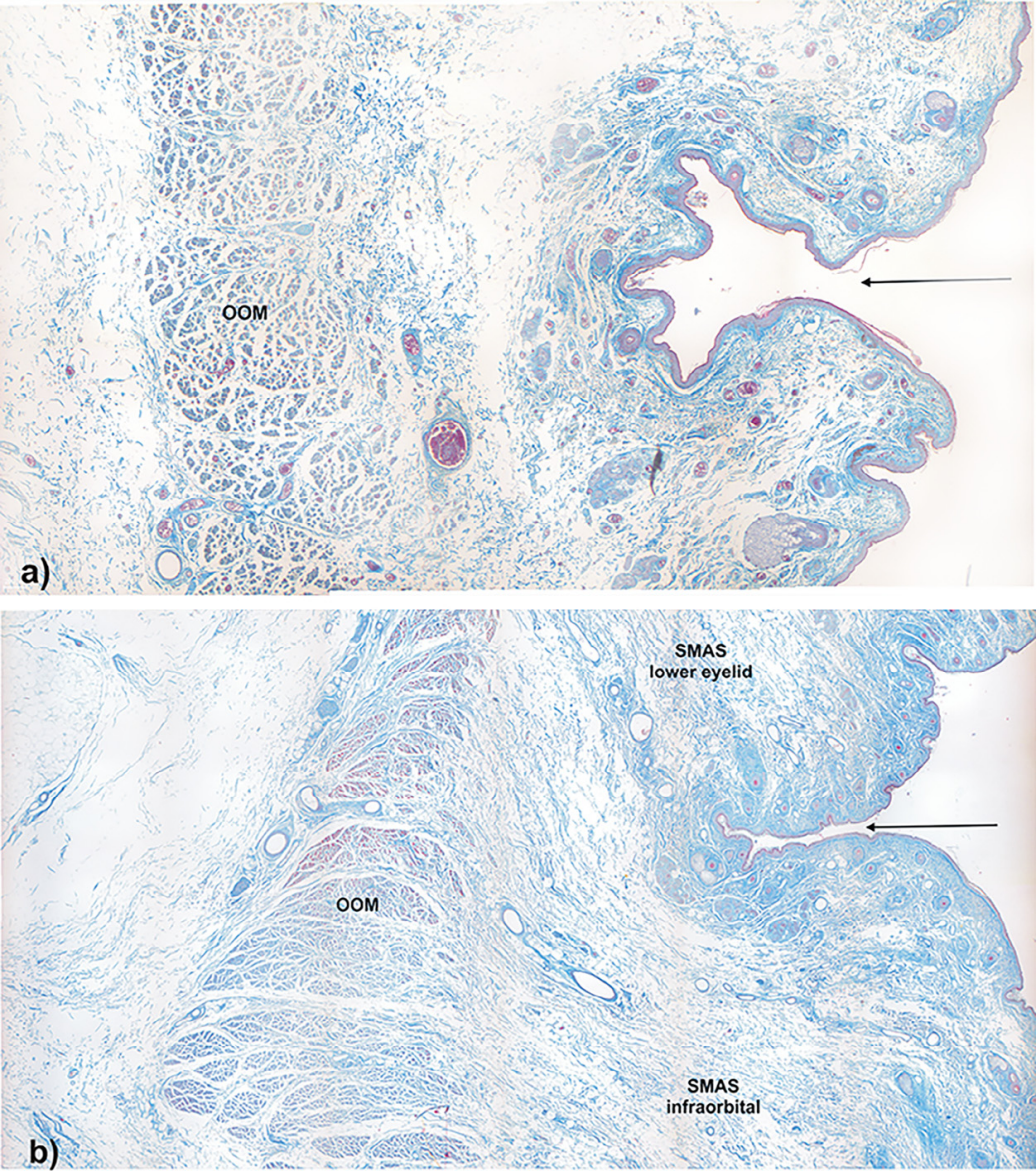
Microphotograph overview of the histological infraorbital fold of the a) specimen one and b) specimen two showing deep cutaneous crinkles.

**Figure 9:**
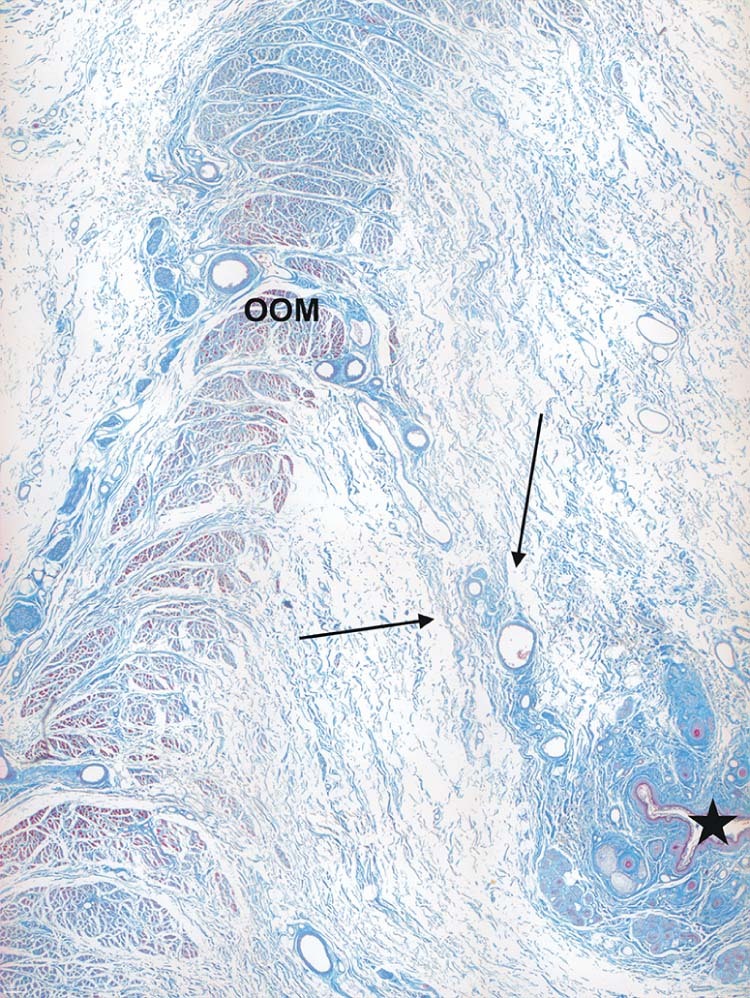
Microphotograph overview of the histological fibro-vascular connection between the OOM and the infraorbital fold (★) in specimen two.

Macroscopically and microscopically, the infraorbital fold was identified as a prominent cutaneous deepening marking the transition zone between the SMAS architectures (Figure 8). The infraorbital fold in specimen two had a suspension to the OOM composed of fibrous septa embracing a multitude of blood vessels (Figure 9). The OOM showed subcutaneous morphological changes without any identifiable fibrous fixation.

**Figure 10:**
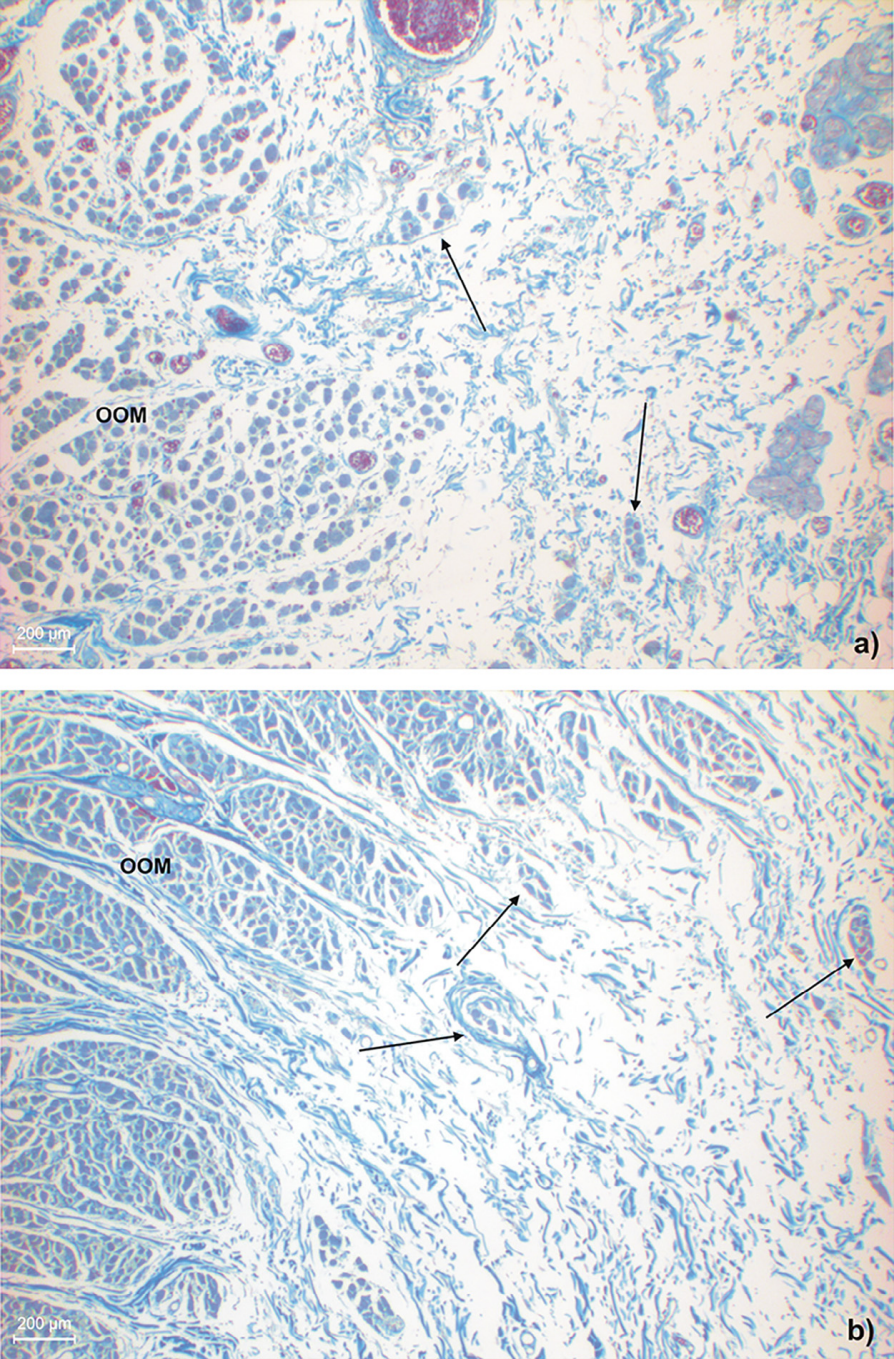
Higher magnification of the SMAS fiber insertion into the OOM in a) specimen one and b) specimen two donors showing muscular bundles along the fibrous septa.

Cranial to the infraorbital fold, the SMAS architecture changed, transforming into irregularly aligned loose connective tissue (Figure 5c and 5d). The infraorbital fold marked the transition zone between the morphological types of SMAS in the infraorbital region. Different SMAS morphologies among all three specimens were observed superior to the infraorbital fold in the lower eyelid region. The SMAS architectures were spotted with isolated muscular bundles. Proximal to the OOM, the density of the muscular bundles increased, leaving the impression that they originated from the OOM (Figure 10).

### 3D reconstruction

**Figure 11:**
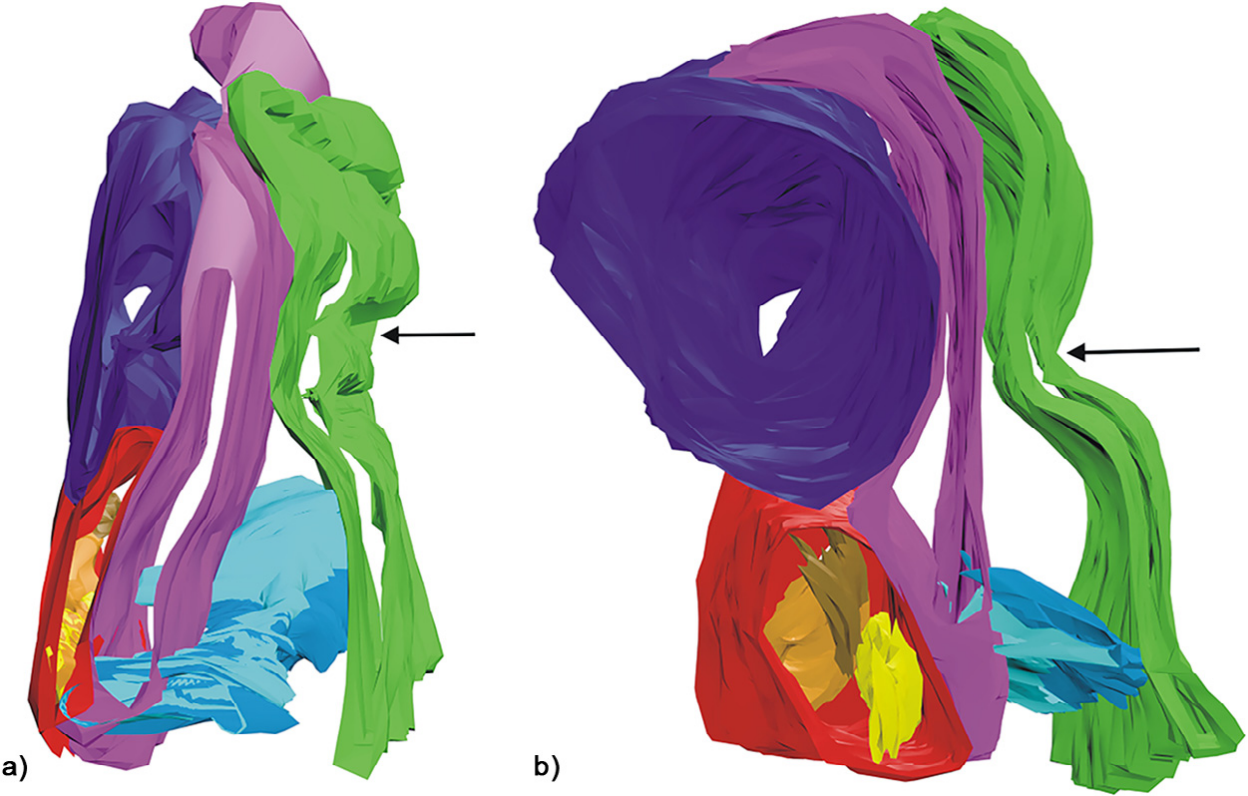
The 3D reconstruction of the a) specimen one and b) specimen two tissue blocks of the inferolateral side of the orbit. Color map: green—skin; violet—intraorbital fat; light blue—SMAS fibers; pink—OOM; red—SOOF; brown, yellow and orange—SOOF fat pads; arrow—infraorbital fold. (For interpretation of the references to color in this figure legend, the reader is referred to the web version of this article.)

The 3D reconstruction simulated the morphological architecture of the tissue blocks of the inferolateral region of the orbit, showing specimen-specific differences of the SOOF volume (Figure 11).

**Figure 12:**
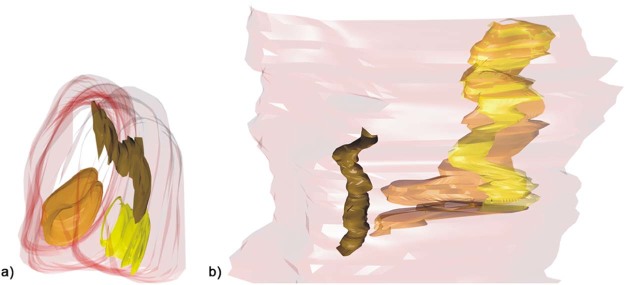
3D reconstruction of the a) specimen one and b) specimen two SOOF fat pads after freezing the shell surrounding the SOOF, demonstrating the connections of the SOOF fat pads. Color map: pink—SOOF shell; brown, yellow and orange—SOOF fat pads. (For interpretation of the references to color in this figure legend, the reader is referred to the web version of this article.)

**Figure 13:**
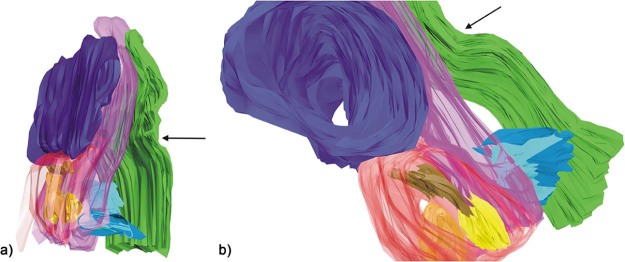
The three-dimensional reconstruction of the a) specimen one and b) specimen two tissue blocks of the inferolateral side of the orbit demonstrating the fibrous connection between the SOOF and SMAS septa crossing the OOM. Color map: green—skin; violet—intraorbital fat; light blue—SMAS fibers; pink—OOM; red—SOOF; brown, yellow and orange—SOOF fat pads; arrow—infraorbital fold. (For interpretation of the references to color in this figure legend, the reader is referred to the web version of this article.)

Processing with AutoCAD revealed that SOOF was a 3D meshwork forming interconnecting spaces similar to SMAS (Figure 12). Similar to the histological analysis, SOOF septa were aligned parallel to the OOM, and the SMAS septa were perpendicularly aligned to the space between the OOM and the skin (Figure 13). The SOOF and SMAS meshwork were connected by fibrous septa perforating and dividing the OOM. There were no direct communicating spaces between the SOOF and the SMAS fat pads.

## Discussion

The results of this study showed that the SOOF is a 3D fibrotic meshwork of communicating spaces enveloping fat pads suspended in the OOM and infraorbital SMAS. Therefore, it can act as a gliding cushion for the OOM. Muscular contractions of the OOM are conducted to the skin by the SMAS while simultaneously mobilizing the SOOF.

According to our results, the SOOF architecture showed fibro-fatty tissue located deep to the OOM, differentiating it from the pure fatty nature of orbital fat and confirming former study results.^2^ The fibrous septa form a 3D meshwork of fatty tissue-filled intercommunicating chambers, which might explain the diffusion effect observed by Rohrich et al. after injecting methylene blue into the sub-OOM plane to dye the SOOF blue.^11^ Rohrich et al. (2009) described a medial and a lateral SOOF compartment.^11^ The 3D reconstruction method used in our study proved that the SOOF is a 3D fibrotic meshwork enveloping fat pads located beneath the OOM. To investigate the existence of the two SOOF compartments, we propose injecting methylene blue into the sub-OOM space prior to the application of the 3D reconstruction method.

These results are supported by recent investigations and demonstrate that the SMAS is the fibro-muscular connective tissue enveloping fat pads connecting the mimic musculature to the skin. Therefore, it spreads in the cheek region lateral to the nasolabial fold superior to the mimic musculature plane without any bony insertions.^5^ Previous studies have used different definitions and morphological descriptions of the structure of the SMAS, which has facilitated the misunderstanding of its localization and topographical anatomy.^4^, ^13^

Our former study demonstrated the existence of two SMAS types bordering the nasolabial fold^5^: SMAS Type I and Type II. SMAS Type I lateral to the nasolabial fold connects the mimic musculature to the skin and consists of a three-dimensional meshwork of communicating fibro-muscular compartments enveloping fat pads. The parallel aligned fibro-muscular septa connect the mimic musculature to the skin, inserting perpendicularly into the corium. SMAS Type II, which is medial to the NLF, represents a condensed irregular meshwork of connective tissue fibers with reduced fat pads and increased fibro-muscular septal density and thickness.

At the infraorbital fold level, the infraorbital SMAS architecture showed similar changes to the nasolabial fold. Beneath the infraorbital fold, the SMAS architecture was identical to the SMAS Type I lateral to the nasolabial fold. The infraorbital fold marked the transition zone, where the SMAS morphology was condensed, losing its regularity and transforming into fibro-elastic connective tissue in the lower eyelid. Therefore, we concluded that there was an analogy between the infraorbital and nasolabial folds, both marking the transition zone where SMAS changed its histological structure. Further, we propose the morphological differentiation of a supplementary SMAS Type III covering the lower eyelid region above the infraorbital fold. The lower eyelid SMAS has a different architecture than Type I and Type II SMAS and can be described as SMAS Type III.

SMAS Type III covering the lower eyelid represents a fat-poor loose fibro-elastic connective tissue with an irregular morphological architecture connecting the OOM to the skin cranial to the infraorbital fold.

In our study, the infraorbital fold marked the transition zone between SMAS Type I and III. Similar changes have been described in former studies, where the nasolabial fold noted the histological change of SMAS morphology from the cheek to the labial side.^5^ In one specimen, there was a supplementary fibro-vascular suspension of the infraorbital fold to the OOM, which supports the hypothesis of the multifactorial genesis of the infraorbital fold. Nevertheless the absence of the fibro-vascular suspension in the other two specimen hypothesized the existence of high inter individual variability of this structure.

SMAS and SOOF showed morphological similarities which supported our hypothesis prior to the investigation. The lack of muscular fibers in the SOOF architecture presumed the different functional interaction of the two tissues to another and to the bordering structures.

As an additional surgical procedure to the lateral tarsal strip, SOOF lifting represents a proper surgical intervention for the rehabilitation of lower eyelid retraction and mid-face ptosis in patients with congenital, Bell's and acquired facial palsy.^6^ Contrary to these results, malar fat repositioning and face lifting surgery have shown short-term success and poor long-term sustainability.^14^, ^15^ A possible explanation could be the thinner atrophic cheek tissue in congenital facial palsy, which could be easily redraped.^6^ Our results have shown a close relationship among SOOF, OOM and the infraorbital SMAS connected by elastic conjunctive tissue. In our opinion, the good long-term results after SOOF lifting in patients with facial palsy are due to the palsy of the OOM, so there is no muscular traction on the suspended SOOF after lifting. Otherwise, in malar, fat and SOOF subperiosteal repositioning procedures,^7^, ^16^, ^17^, ^18^ mimic muscular contractions are transferred via the connective tissue to the SOOF, drifting it to the initial ptosis position.

The results of this study might be beneficial to the clinical practitioner in improving the long-term postoperative effects for periocular surgical procedures and midfacial rejuvenation techniques.

## Conclusions

SOOF is a fibro-fatty tissue pad on the inferolateral orbital border with variable size.

SOOF and SMAS have histomorphological and architectural similarities and are connected by fibrous septa that divide the OOM into a variety of muscular bundles. The direct SOOF connection to the OOM can be interpreted as the cause of the poor long-term results after SOOF lifting. SMAS Type III covers the lower eyelid region superiorly to the infraorbital fold. The infraorbital fold originates due to a morphological change of the SMAS architecture and a fibro-vascular suspension.

## Conflict of interest

The authors declare that they have no conflict of interests.

## Appendix Supplementary data

Video S1 Video of a rotating tissue block of a 3D reconstruction of a female body donor. The colors assigned to the structures are corresponding with those of Fig. 11. Video S1

Video S2 Video of a rotating tissue block of a 3D reconstruction of a male body donor. The colors assigned to the structures are corresponding with those of Fig. 13. Video S2
